# Prevalence, types, maternal and perinatal outcomes of cardiac disease in pregnancy at a tertiary Hospital in the Eastern Cape, South Africa

**DOI:** 10.4102/jcmsa.v4i1.274

**Published:** 2026-09-12

**Authors:** Sibusiso B. Mbili, Vulikhaya Mpumlwana, Charles B. Businge

**Affiliations:** 1 Department of Obstetrics and Gynaecology, Faculty of Medicine and Health Sciences, Walter Sisulu University, Mthatha, South Africa; 2 Department of Obstetrics and Gynaecology, Faculty of Health, Nelson Mandela Academic Hospital, Mthatha, South Africa

**Keywords:** cardiac disease in pregnancy, rheumatic heart disease, obstetrics, peripartum cardiopulmonary, maternal and foetal outcomes

## Abstract

**Background:**

Cardiac disease is a leading non-obstetric cause of maternal morbidity and mortality, particularly in low- and middle-income countries.

**Methods:**

Retrospective cross-sectional review of all deliveries from 01 January 2020 to 31 December 2022. Echocardiography-confirmed cardiac disease cases were identified. Data included demographics, lesion type and/or severity (New York Heart Association [NYHA]), timing of diagnosis, delivery characteristics and outcomes. Associations were examined using χ^2^ and *t*-tests (*p* < 0.05).

**Results:**

Among 11 728 deliveries, 182 had cardiac disease (prevalence 1.6%, 95% CI: 1.34–1.80). Median age was 26 years (IQR 22–31); 50.6% presented with NYHA III–IV. Rheumatic heart disease (RHD) predominated (61.5%), mainly mitral stenosis (43.8%) and mitral regurgitation (38.3%). Cardiomyopathy accounted for 31.3%; peripartum cardiomyopathy (PPCM) comprised 78.9% of cardiomyopathies, most diagnosed postpartum (64.9%) and frequently with Left Ventricular Ejection Fraction (LVEF) < 45%. Preterm birth occurred in 46.9% of pregnancies, particularly with RHD–MS (mean gestational age (GA) 33.0 ± 6.0 weeks; *p* = 0.005). Caesarean delivery occurred in 72.0% (131/182), mainly for obstetric (41.8%) rather than cardiac indications. Maternal complications were common (67.6%), including pulmonary oedema (28.6%, 95% CI: 22.1–35.7), pulmonary hypertension (13.2%, 95% CI: 8.6–19.0) and ICU admission (12.1%, 95% CI: 7.8–17.6). Maternal case fatality was 4.9% (9/182; 95% CI: 2.3–9.2). Most neonates had favourable short–term outcomes (5–min Apgar ≥ 7 in 83.5%) and were discharged (87.7%).

**Conclusion:**

Rheumatic heart disease and PPCM were the dominant lesions and major drivers of severe morbidity and mortality.

**Contribution:**

The high burden of late presentation and severe functional limitation underscores the need for earlier detection, systematised cardio-obstetric care and strengthened primary prevention of RHD.

## Introduction

Cardiac disease in pregnancy is a major problem worldwide, particularly in the low- and middle-income countries (LMICs).^1^ Cardiac disease in pregnancy is the most important non-obstetric cause of maternal death and is associated with significant maternal and neonatal morbidity and mortality, though the mortality has now reduced.^1,2,3^ Cardiac disease continues to be a significant cause of death 6 weeks postpartum in LMICs.^4^ The incidence of cardiac disease in pregnancy varies worldwide and is between 0.1% and 4%.^1,5^ Sub-Saharan Africa bears a disproportionately high burden of rheumatic heart disease (RHD) among women of reproductive age.^6^ South Africa estimates range from 5 to 15.^2,4,7^

Congenital heart disease is the most frequent cardiovascular disease present during pregnancy in high-income countries, while RHD is predominant in LMICs like South Africa.^8,9^ Peripartum cardiomyopathy (PPCM) is the common cause of severe complications and is characterised by significant left ventricular dysfunction due to the enlargement and subsequent weakening of the heart muscles.^1^ More and more women are getting pregnant with pre-existing cardiac disease.^2,10^ Pregnancy can worsen any established cardiac disease, and cardiac disease can manifest itself for the first time during and after pregnancy.^11^

Despite the reported high incidence of maternal and perinatal morbidity and mortality from cardiac disease in pregnancy, there is a paucity of local literature regarding the prevalence, main contributing types and associated clinical features and lastly, maternal and perinatal outcomes of cardiac disease in pregnancy in the Eastern Cape province and other rural settings in South Africa. According to the recent 2020–2022 Saving Mother’s report, pre-existing medical and surgical conditions remain the third most common direct cause of maternal mortality in the Eastern Cape, accounting for 17.64% of maternal deaths, with cardiac disease accounting for one-third of this. However, the maternal and perinatal outcomes of patients with cardiac disease in the north-eastern part of the Eastern Cape have not yet been documented; the major cardiac lesions and their associated maternal and foetal outcomes are not known with certainty.

## Research methods and design

### Study design and setting

We conducted a retrospective cross-sectional study at Nelson Mandela Academic Hospital (NMAH), a tertiary hospital that receives referrals from O.R. Tambo, Alfred Nzo and parts of Chris Hani districts, Eastern Cape, South Africa, from 01 January 2020 to 31 December 2022. A consecutive sampling method was used until the required number of participants had been recruited.

### Participants

We included all echocardiography-confirmed cardiac disease cases in pregnancy or ≤ 6 weeks postpartum. We excluded records without echo confirmation.

### Variables and definitions

Lesions are categorised as RHD (Mitral Stenosis [MS], Mitral Regurgitation [MR], Mixed Mitral Valve Disease [MMVD]), cardiomyopathy (PPCM defined per the European Society of Cardilogy [ESC] criteria), congenital heart disease and diastolic dysfunction secondary to hypertensive disorders. Severity is graded by New York Heart Association (NYHA). Left Ventricular Ejection Fraction (LVEF) is obtained from standard transthoracic echo.

*Severe maternal outcome* is defined according to WHO criteria as either maternal death or a near-miss. A maternal near-miss is defined as a woman who nearly died but survived a life-threatening complication occurring during pregnancy, childbirth or within 42 days of termination of pregnancy. Near-miss cases were identified using the WHO organ dysfunction-based criteria, which included cardiovascular, respiratory, renal, coagulation/haematological, hepatic, neurological or uterine dysfunction.

*Maternal cardiac complication* is defined as the occurrence of one or more serious cardiovascular events during pregnancy, delivery or postpartum period, including cardiac decompensation requiring treatment or hospitalisation, pulmonary oedema, clinically significant arrhythmias requiring intervention, thromboembolic events, cardiac arrest and the need for Intensive Care Unit (ICU) admission for a cardiac indication.

*Perinatal complication* is defined as adverse foetal or neonatal outcomes occurring during pregnancy or within the first seven completed days after birth. It includes preterm birth (< 37 completed weeks), low birth weight (< 2500 g), Apgar score < 7 at 5 min, Neonatal Intensive Care Unit (NICU) admission, stillbirth (foetal death at ≥ 28 weeks of gestation or according to the national definition), early neonatal death (within the first 7 days of life), foetal growth restriction and respiratory distress syndrome where applicable.

### Statistical analysis (augment)

Continuous variables were summarised as mean ± s.d. for normally distributed data or median (interquartile range [IQR]) for non-normally distributed data. Categorical variables were summarised as frequencies and percentages (*n/N*, %). Comparisons between groups were performed using the Pearson chi-square test or Fisher’s exact test for categorical variables and the independent samples *t*-test or Mann–Whitney *U* test for continuous variables as appropriate. Univariate logistic regression analyses were performed to evaluate the association between each primary outcome (preterm birth, ICU admission, Apgar score < 7 at 5 min and maternal mortality) and the variables of interest including age, parity, booking status, anaemia, lesion group, NYHA functional class and timing of diagnosis. Results are reported as odds ratios (ORs) with 95% confidence intervals (95% Cis). Statistical significance was set at *p* < 0.05. Analyses used SPSS v24.0.

### Ethical considerations

Ethical clearance to conduct this study was obtained from the Walter Sisulu University Research Ethics Committee on 19 July 2023 (No: 116/2022) before commencement of the study. Permission to carry out research was also sought from the administration of NMAH and the Eastern Cape Department of Health (No: EC_202307_014).

## Results

There were 11 728 deliveries at NMAH during the study period. Of these, 182 were complicated by cardiac disease and were all included in the current study, thus resulting in a prevalence of 1.6%.

### General characteristics

The mean age was 27.1 ± 7.3 years with a range of 14–47 years. Most of the participants (125/182, 68.7%) were in the age category of 20–34 years. Seventeen per cent (31/182) had advanced maternal age (≥ 35 years), and 14.3% (26/182) were teenagers. Of the 182 participants, 66 (36.3%) were primigravidae, 46 (25.3%) gravida 2, 33 (18.1%) gravida 3 and 37 (20.3%) gravida 4 or more. Only 72.5% (132/182) were booked. These included 23 of 25 (92%) participants with known cardiac lesions before the index pregnancy and 109 of 157 (69%) who were only diagnosed with cardiac disease during the index pregnancy. The overall mean gestational age (GA) at the first antenatal booking was 21.9 ± 6.2 (range of 8.1–39.2 week of emenorrhoea [WOA]) (Table 1).

**TABLE 1:** Demographics and clinical characteristics of mothers with cardiac disease at Nelson Mandela Academic Hospital.

Characteristic	*n*	%
**Age category (years)**		
≤ 19	26	14.3
20 to < 35	125	68.7
≥ 35	31	17.0
**Gravidity**		
Primigravida	66	36.3
Gravida 2	46	25.3
Gravida 3	33	18.1
Gravida ≥ 4	37	20.3
**Haemoglobin (g/dL)**		
Normal (> 11)	110	60.4
Mild anaemia (10.1–10.9)	57	31.3
Moderate anaemia (7.0–9.9)	15	8.2
**Booking status**		
Booked	132	72.5
Unbooked	50	27.5
**District**		
Amathole	14	7.7
Alfred Nzo	37	20.3
Chris Hani	6	3.3
Joe Gqabi	7	3.8
O.R Tambo	118	64.8
**Health facility referred from**		
District hospitals	136	74.7
Local clinics	19	10.4
Private specialist	27	14.8

**Total**	**182**	**100.0**

Those who were diagnosed with cardiac disease during the index pregnancy were booked at a gestational age of 22.2 ± 6.0 weeks, and the difference between the two groups was not statistically significant (*p* = 0.216). A hundred and ten participants (60.4%) had normal Hb at booking, 57 (31.3%) had mild anaemia, and 15 (8.2%) had moderate anaemia. None had severe anaemia. All of the 182 patients with cardiac disease enrolled in the current study were referred from the following facilities: 74.7% were referrals from district hospitals, 10.4% from local clinics and 14.8% from private specialists’ facilities. Most of the patients were from OR Tambo (64.8%) and Alfred Nzo districts (20.3%) (Table 1).

### Maternal characteristics

#### Timing of diagnosis of cardiac lesion

At the time of admission to NMAH, about a third (62/182, 34.1%) had a confirmed diagnosis of cardiac disease from the referring health unit, including the 25 participants (13.7%) who were diagnosed before the index pregnancy. Twelve (12/25) had had cardiac surgery before the index pregnancy, although all had defaulted from follow-up.

The rest (120/182, 65.9%) presented with various symptoms and signs of respiratory distress, with most initially diagnosed as pulmonary oedema (52/182, 28.6%).

Most participants were diagnosed with cardiac disease during the antenatal period (86/182, 47.3%) and in the postpartum period (70/182, 38.5%). Only one was diagnosed during labour (0.5%) (Table 2).

**TABLE 2:** Timing of diagnosis of the cardiac lesions.

Time	*n*	%
Pre-pregnancy	25	13.7
Antenatally	86	47.3
Intrapartum	1	0.5
Post-partum	70	38.5

#### Frequency and type of cardiac lesion

Most of the participants (112/182, 61.5%) had RHD. Fifty-seven (31.3%) had cardiomyopathy. Of the 57 participants with cardiomyopathy, 45 (78.9%) had PPCM. Seven (7/182, 3.8%) had congenital heart lesions (five with Tetralogy of Fallot and two with ventricular septal defects), and six (6/182, 3.3%) had diastolic dysfunction secondary to hypertensive disease in pregnancy. All the participants with congenital lesions had had cardiac surgery, with only two of these having residual disease during the index pregnancy (Table 3).

**TABLE 3:** Type and prevalence of cardiac lesions.

Type of lesion	*n*	%
Cardiomyopathy	57	31.3
Congenital heart disease	7	3.8
Hypertensive cardiac disease	6	3.3
Rheumatic heart disease	112	61.5

**Total**	**182**	**100.0**

#### Association between age and type of cardiac lesion

The age of presentation for patients with cardiomyopathy was significantly higher than for other cardiac lesions (30.3 ± 7.7, *p*-value = 0.004) (Table 4).

**TABLE 4:** Maternal age according to cardiac lesions.

Lesion	*n*	%	Mean age	Median	Range	*p*-value*
CM	57	31.3	30.3 ± 7.7	31.0	15–47	0.004
CHD	7	3.8	24.0 ± 3.5	24.0	20–29	-
DD-HDP	6	3.3	24.3 ± 7.8	22.0	18–39	-
RHD MS	49	26.9	25.9 ± 7.4	24.0	14–46	-
RHD MR	43	23.6	26.2 ± 6.8	25.0	15–38	-
RHD-MMVD	20	11.0	24.9 ± 5.5	25.5	15–34	-

**Total**	**182**	**100.0**	**-**	**-**	**-**	**-**

CM, Cardiomyopathy; CHD, congenital heart disease; DD-HDP, Diastolic Dysfunction Hypertensive Disorder of Pregnancy; RHD MS, Rheumatic Heart Disease with Mitral Stenosis; RHD MR, Rheumatic Heart Disease with Mitral Regurgitation; RHD MMVD, Rheumatic Heart Disease with Mixed Mitral Valve Disease; ANOVA, Analysis of Variance.

* , *p*-value for ANOVA.

#### Types of rheumatic heart lesions

Of the 112 participants with RHD, 49 (43.8%) had MS, 43 (38.3%) MR and 20 (17.9%) MMVD (Figure 1).

**FIGURE 1:**
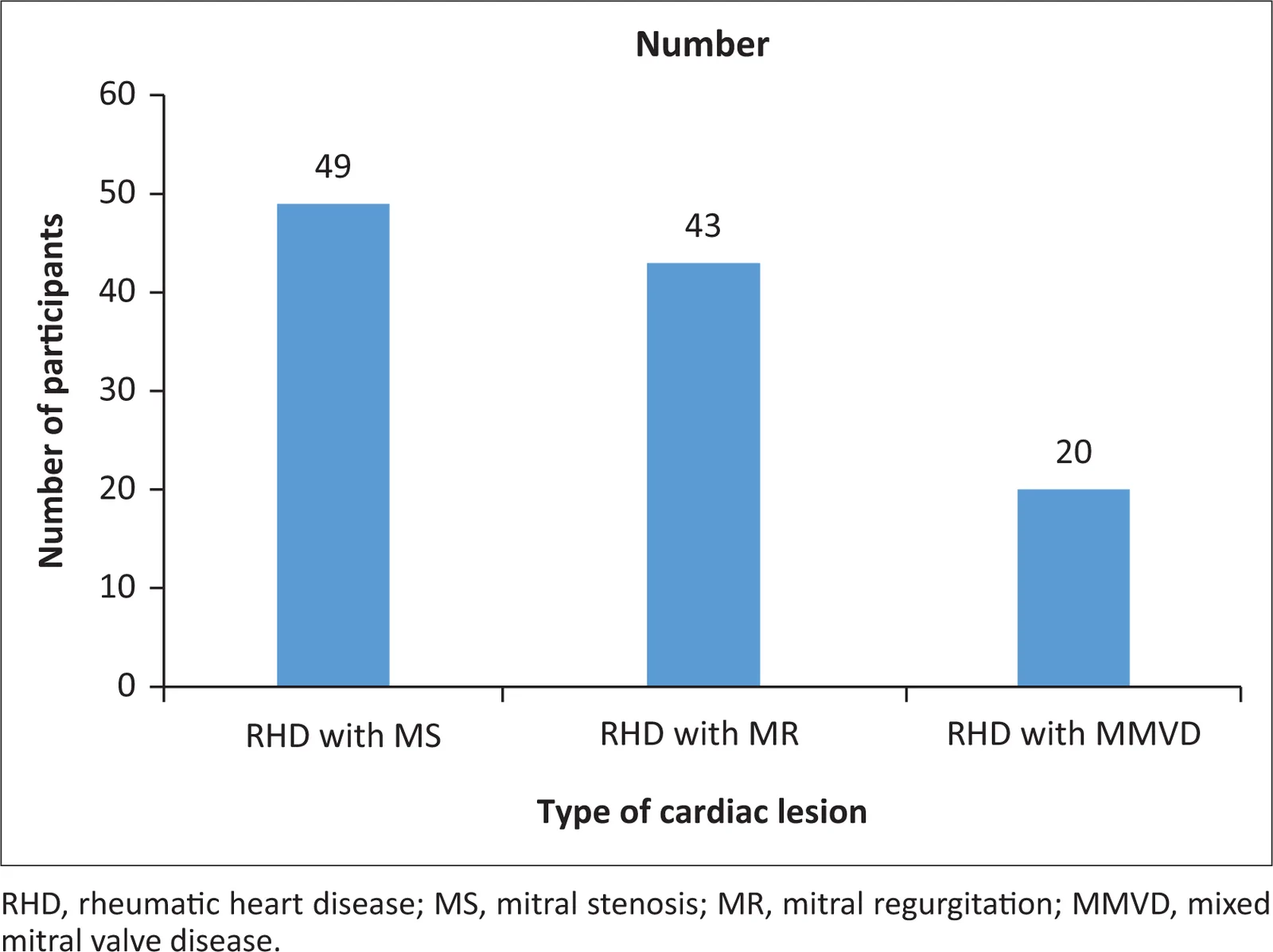
Types of rheumatic heart lesions of the participants.

#### Left ventricular ejection fraction of participants with cardiomyopathy

Fifty-seven participants (31.3%) were diagnosed with cardiomyopathy. Most of them 45/57 (78.9%, 95% CI: 88.1–88.6) had PPCM with severe left ventricular dysfunction (LVEF ≤ 45%) (Table 5). Of all the participants with PPCM, diagnosis most commonly occurred after delivery (*n* = 37, 64.9%). Twenty-eight of these participants had delivered at the referring health units, while seven delivered following referral to NMAH.

**TABLE 5:** Left ventricular ejection fraction of participants with cardiomyopathy.

Ejection fraction	*n*	%
< 45% (PPCM)	45	78.9
≥ 45% (other cardiomyopathies)	12	21.1

**Total**	**57**	**100.0**

PPCM, Peripartum Cardiomyopathy.

#### Functional status at referral

Participants with more severe cardiac disease, NYHA III (66/182) and IV (26/182), comprised more than half of the entire sample (50.6%) (Figure 2).

**FIGURE 2:**
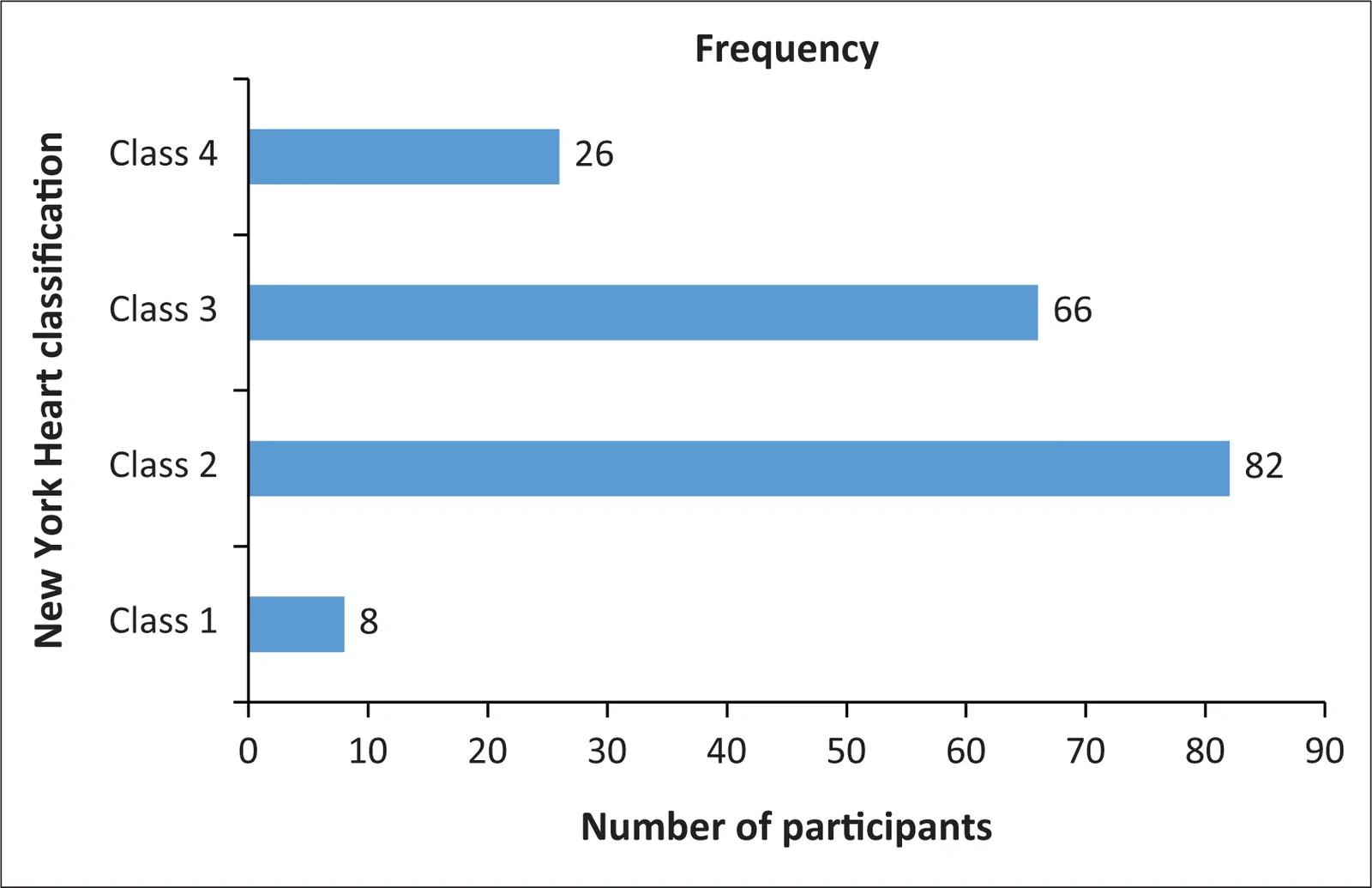
Bar graph of New York Heart Class of participants at the time of admission.

### Maternal outcomes

#### Time of delivery

The majority of women delivered at term (45.6%), followed by those who delivered late preterm (31.3%). About 6% had miscarriages or their pregnancies were terminated ≤ 26 WOA. Participants with RHD complicated by MS delivered significantly earlier than all the other women (mean gestational age at delivery 33.0 ± 6.0 WOA, *p* = 0.005).

#### Mode of delivery

There was no significant difference in the mode of delivery of the participants with various categories of cardiac lesions. Overall, 50.5% were delivered by emergency caesarean section, followed by elective caesarean section (21.4%) and spontaneous vaginal delivery (18.9%). Only 8.3% delivered following induction of labour (IOL) (*p* = 0.146).

Most participants delivered through caesarean section (71.9%; *n* = 131). Of these, the major indications were obstetric causes, which included foetal distress, two or more previous caesarean sections, and previous caesarean sections with preeclampsia or other medical conditions (41.8%; *n* = 76), followed by a combination of obstetric and cardiac complications (26.9%; *n* = 49). Only nine (4.9%) were delivered solely due to cardiac complications. The majority of participants who delivered vaginally (26.4%; *n* = 48) or by caesarean section (41.8%, *n* = 76) had no cardiac decompensation but obstetric indications at the time of delivery.

#### Maternal complications

Almost 70% of the mothers experienced complications (*n* = 123; 67.6%). The most common complications were pulmonary oedema (*n* = 52, 28.6%) and severe pulmonary hypertension (*n* = 24, 13.2%) (Table 6).

**TABLE 6:** Other maternal complications.

Complication	*n*	%	95% CI
Maternal mortality	9	4.9	2.3–9.2
ICU admission	22	12.1	7.8–17.6
Pulmonary oedema	52	28.6	22.1–35.7
Pulmonary hypertension	24	13.2	8.6–19.0
New onset AF	4	2.2	0.6–5.5
Thromboembolic event	2	1.1	0.1–3.9
Heart failure	10	5.5	2.7–9.8

AF, atrial fibrillation; CI, confidence interval; ICU, intensive care unit.

#### Admission to the intensive care unit

There were 22 ICU admissions (12.1%, 95% CI: 7.8–17.6). There was no significant difference in ICU length of stay according to cardiac lesions. However, cardiomyopathy accounted for 54.5% (*n* = 12) of the participants who were admitted to the ICU.

#### Maternal mortality

Nine of the 182 participants demised, giving a case-fatality rate of 4.9% (95% CI: 2.3–9.2). Pulmonary hypertension and pulmonary oedema were the most common complications preceding maternal death. These occurred mostly in patients with RHD, especially among patients with NYHA III–IV (Table 7).

**TABLE 7:** Maternal death profile (*N* = 9).

Characteristics	*n*
**Booking status**	
Booked	6
Unbooked	3
**Time of death**	
Antenatally	4
Intrapartum	1
Postpartum	4
**Type of cardiac lesion**	
Peripartum cardiomyopathy	1
RHD with MS	5
RHD with MR	1
RHD with MMVD	2
**New York Heart Association Class**	
NYHA class I–II	1
NYHA class III–IV	8
**Contributing complications**	
Pulmonary oedema	3
Pulmonary hypertension	5
**Neonatal outcome**	
Alive	4
Stillbirth	1
Undelivered	4

RHD MS, Rheumatic Heart Disease with Mitral Stenosis; RHD MR, Rheumatic Heart Disease with Mitral Regurgitation; RHD MMVD, Rheumatic Heart Disease with Mixed mitral valve disease; NYHA, New York Heart Association.

### Perinatal outcomes

#### Apgar score

Most babies (83.5%; *n* = 152) born to mothers with cardiac disease had 5-min Apgar score ≥ 7 despite the maternal condition. However, significantly more babies with a 5-min Apgar score < 7 or fresh stillbirth (FSB) and macerated stillbirth (MSB) were born to mothers with either PPCM or RHD with MS (*p* = 0.035). Almost 90% of the births were live (85.7%; *n* = 156).

#### Birth weight

Most babies (57.7%; *n* = 105) had a normal birth weight of 2501 g – 4000 g. There was no significant difference in birth weight according to the type of cardiac condition of the mother (*p* = 0.428). Four mothers died undelivered.

#### Overall neonatal condition

All babies born to mothers with either congenital cardiac lesions or diastolic dysfunction secondary to hypertensive heart lesions were alive and stable at birth and did not require neonatal admission or any therapeutic interventions before they were discharged. Most babies born to mothers with other lesions, however, were also stable at birth.

## Discussion

### Prevalence of cardiac disease

The prevalence of cardiac disease in pregnancy at NMAH in this study was found to be 1.6%. This prevalence falls within the worldwide prevalence of cardiac disease in pregnancy of 1%–4%.^2,5,12^ This is comparable to findings by Shrestha et al.^13^, who in a systematic review of studies from South-east Asia found a prevalence of 1.5%, and Beaton et al.^14^, who found a prevalence of 1.7% in Uganda.

The results of the current study, however, differ from those reported by Soma-Pillay et al.^8^, who found a prevalence of only 0.9% in a study carried out at Pretoria Academic Hospital and Balieva et al.^1^, who found a prevalence of 0.8% at Charlotte Maxeke Johannesburg Academic Hospital. Owens et al.^15^, in a study done in the United States (US), found a prevalence of only 0.2%. The observed similarities and differences between the findings of the current study and the various studies can be explained by differences in patient populations and access to diagnosis and care of heart disease in pregnancy. Rheumatic heart disease is the predominant cardiac disease seen in pregnant women in low-resource settings, while it is rare in high-income countries.^16^

### General population characteristics

#### Age of the participants

The mean age of the pregnant women with heart disease in this study was 27.1 ± 7.3 years (range of 14–47 years). This is comparable to 27.4 ± 6.7 years reported by Soma-Pillay et al.^8^ in Pretoria, and a median age of 26 (16–50) reported by Lumsden et al.^6^ in a study from western Kenya. However, the mean age of mothers with cardiac disease in this study is lower than that reported by others. Owens et al.^15^ in the US Statewide Planning And Research Cooperative System (SPARCS) study found the mean age to be 29.3 ± 6.3 years, whereas Silversides et al.^17^ who found a mean age of 30.6 ± 5.6 years in a study conducted in Canada at two large tertiary care hospitals, and Balieva et al.^1^ found a mean age of 30.2 ± 6.4 years among patients receiving care at a tertiary hospital in Johannesburg, South Africa.

Most studies from high-income settings report a higher mean age among mothers with cardiac disease in pregnancy than the one found in the current study. This can be explained by the early age of childbearing that is common in low-resource settings, which inevitably unmasks silent cardiac disease due to the physiological changes of pregnancy.^18^ This is confirmed in the current study by the result that shows that 86.3% of the participants were diagnosed with cardiac disease during the index pregnancy.

#### Gravidity and parity

Whereas in high-resource countries, more than 50% of patients with cardiac disease in pregnancy are primigravidae,^19^ and the current study found that primigravida comprised only 36.3% of the mothers. This is similar to findings of studies from other low-income settings, such as Lumsden et al.^6^ in a study done in western Kenya, a proportion of 36.1%, and Soma-Pillay et al.^8^, a proportion of 34.4% at a tertiary hospital in Pretoria, South Africa. The differences in the gravidity of patients with heart disease in low-resource and high-resource settings are due to both higher age at initial childbearing and low parity in high-income settings.

#### Antenatal care attendance and haemoglobin

More than a quarter (27.5%) of the mothers with cardiac disease in this study had not initiated antenatal care and presented for the first time with cardiac decompensation. The mean gestational age at the first antenatal visit (booking) for the remainder (72.5%) was 21.9 ± 6.2 WOA (range of 8.1–39.3). This is comparable with the findings of Makgato^18^ in a study done at Universitas Academic Hospital in Bloemfontein, South Africa, where the mean gestational age at booking was found to be 19.8 WOA. This is because most of the patients were asymptomatic, had never been diagnosed with cardiac disease (86% in the current study), and, like most pregnant women in South Africa, presented in the second trimester.^20^

While anaemia in pregnancy can unmask previously undiagnosed or worsen the status of women with cardiac disease, most of the participants in our study had optimum haemoglobin levels. This may be accounted for by the low prevalence of anaemia in pregnancy, which has been previously reported to be less than 19% at booking.^21,22^

#### Timing of diagnosis of cardiac disease

In the current study, only 13.7% (*n* = 25) of participants had a known cardiac lesion pre-pregnancy, while 47.3% (*n* = 86) were diagnosed during the antenatal period and 38.5% (*n* = 70) were diagnosed postpartum. This contrasts with the findings of Diao et al.^23^ in a study conducted at the Cardiology Department of the University Hospital in Dakar, Senegal, in which pre-pregnancy diagnosis of cardiac disease occurred in 74% of cases. This can be attributed to the more severe disease in the study by Diao et al. (NYHA III or IV of 94.1%) compared to the current study (NYHA III or IV of 50.5%). This could also be due to a higher level of surveillance for cardiac disease at the University Hospital in Dakar, which is an urban setting compared to the more rural setting around NMAH. Furthermore, in the current study, there was a disproportionately high incidence of PPCM whose risk factors warrant further study.

#### Distribution of participants by districts

Nearly two-thirds of participants were from O.R Tambo district. This can be explained by the fact that O.R Tambo district has the largest concentration of population in the Eastern Cape province, with 1 501 702 people, followed by Nelson Mandela Bay with 1 190 496 people (Census 2022). Nelson Mandela Academic Hospital is a tertiary hospital that gets referrals mainly from O.R Tambo and Alfred Nzo districts; very few come from other districts such as Joe Gqabi, Amathole and Chris Hani.

#### Distribution of participants by health facility referral type

The majority of the participants referred to NMAH were coming from district hospitals. According to the hospital classification of the Western Cape Government, district hospitals have limited specialist services, while academic hospitals have the most specialist services.

### Maternal characteristics

#### Distribution of participants’ cardiac disease diagnoses

The most common cardiac disease in pregnancy at NMAH was RHD, mostly characterised by MS and MR. A study from Tygerberg Hospital in South Africa reported similar findings, with RHD as the leading heart condition in pregnancy, with the most common abnormalities being MR and MMVD.^24^ Similarly, in a study from Johannesburg, RHD was the most common heart condition seen in pregnancy (28.4%).^1^ An earlier retrospective chart review in South Africa had also found RHD was the leading heart condition seen in pregnancy.^7^ A subsequent systematic review from South Africa found that RHD with MS, RHD with MR, and prior surgical mitral valve repair accounted for 71.8% of antenatal heart disease.^25^

Similar findings have also been reported from elsewhere in sub-Saharan Africa. In Eritrea, the common cardiac disease was also RHD.^26^ Similarly, a Kenyan study found RHD to be the most common abnormality of cardiac disease (75%), and more than half of those were complicated by either severe MS or pulmonary hypertension.^6^

According to French and Poppas, ‘RHD is a disease of poverty’ as rheumatic fever and RHD have almost been completely eradicated in the developed world.^27^

The prevalence of RHD in our cohort likely reflects the persistent burden of socioeconomic disadvantage in rural South Africa. Poverty, household overcrowding and limited access to primary healthcare contribute to recurrent untreated Group A streptococcal infections during childhood, increasing the risk of acute rheumatic fever and subsequent chronic valvular heart disease. In addition, delayed diagnosis, suboptimal adherence to long-term benzathine penicillin prophylaxis and limited access to specialist cardiology services may allow progression of valvular lesions before pregnancy. Access to definitive interventions, including percutaneous balloon mitral valvotomy and valve replacement surgery, remains limited for many women in resource-constrained settings. Consequently, many present during pregnancy with advanced cardiac disease and significant functional impairment.

#### Time of participants’ diagnosis with cardiac disease

About a third of the women were diagnosed before referral, whereas the rest presented with symptoms of respiratory distress and were diagnosed during the antenatal and postpartum periods. Similar to our findings, the study from Western Kenya found that diagnosis of maternal cardiac disease occurred late, with more than half of the women being diagnosed either during the antenatal or postpartum periods.^6^ In the current study, after RHD, the second most common cardiac lesion was PPCM, which, according to pathophysiology, tends to present late in pregnancy. More than 60% of the participants with PPCM in our study presented in the postpartum period. This correlated well with findings by Sliwa et al.^28^, who in a study conducted in 49 countries, reported that 67% of the participants with PPCM presented after delivery.

#### Distribution of maternal age according to cardiac lesions

The majority of the participants (about 65%) were below 30 years and presented with complications of RHD diagnosed for the first time in the index pregnancy. This is in keeping with findings of previous systematic epidemiological studies that reported about 8%–28% of cardiac disease was unmasked by the hemodynamic stresses of pregnancy that are characterised by increased stroke volume, heart rate and cardiac output.^25,27^ On the other hand, most of the remaining 35% of the participants (*n* = 57, 31.3%) had cardiomyopathy (*n* = 45/57, 78.9% PPCM) and tended to be older than 30 years. This result concurs with a study done by Owens et al.^15^ in the US, who reported a mean age of women with PPCM of 30.3 ± 6.4 years. This is expected as the prevalence of PPCM increases after 30 years of age^29^, with the majority of patients being diagnosed in the postpartum period.^28^

#### Prevalence of severe cardiac disease

More than half (50.6%) of the women in the current study presented with severe cardiac disease (class III and IV of the NYHA classification). This is slightly less compared to the findings from a similar study in Western Kenya that found a prevalence of severe cardiac disease (NYHA III or IV) of 61.4%.^6^ These high percentages imply an unmet need for early diagnosis and management of cardiac disease or contraception in these two rural settings of western Kenya and Eastern Cape, South Africa. In contrast, studies done in urban settings of Johannesburg, South Africa, reported lower prevalences of severe cardiac disease in pregnancy, only 9.5% and 25.3%^1,7^, while a study done in Canada by Silversides et al.^17^ found a much lower prevalence of 2.4%.

This disparity in severe cardiac disease may be explained by low socio-economic status and lack of access to adequate medical services capable of preventing and early treatment of rheumatic fever and early diagnosis and management of RHDs in terms of prevention of recurrence of rheumatic fever, which is responsible for worsening of rheumatic heart lesions. This can be prevented by prophylactic penicillin.^30^ The other aspect of early diagnosis and management is in terms of valvotomy and valve replacement surgery. Furthermore, the lack of health education common in low-resource settings may partially explain the delay in cardiac disease diagnosis and severity of the conditions at presentation.

#### Left ventricular ejection fraction of participants with cardiomyopathy

In the current study, almost 59.6% (34/57) of the participants with cardiomyopathy (*n* = 45, 78.9% PPCM) had an LVEF of < 45%, the majority of whom (*n* = 37, 64.9%) presented in the post-partum period, consistent with its recognised clinical presentation. This is in contrast with the results from the study done by Lumsden et al.^6^ in Western Kenya, who found a rate of left ventricular dysfunction < 45% of only 9.8%. However, Lumsden et al.^6^ estimated the rate of LVEF < 45% in the entire population of women with cardiac disease in pregnancy, which accounts for the observed difference between their study and the current study.

A recent systematic review and meta-analysis conducted on women with cardiomyopathy found that the LVEF of these women was significantly lower than that of women without heart disease (mean difference: –25.17, 95% CI: –38.98 to –11.35, *p* < 0.0004).^31^ These findings and those from our study can be explained by the aetiology of PPCM, which is characterised by significant left ventricular dysfunction due to the enlargement and subsequent weakening of the heart muscles.^28,29^ HIV infection remains prevalent in South Africa and has been implicated as a potential contributor to myocardial dysfunction in susceptible women. Nutritional deficiencies and inflammatory mechanisms have also been proposed, although supporting evidence remains inconsistent.

### Maternal outcomes

#### Gestational age distribution at delivery

More than half of the participants (52.9%) had their pregnancies ending prematurely, with 6% in the pre-viable gestational period and 46.9% having preterm births. This mainly affected women with RHD complicated by MS. The rate of preterm delivery in the current study was much higher than the 33.3% reported by Nqayana et al.^7^ in a study conducted at a tertiary hospital in Johannesburg, South Africa. The difference between the two studies could be attributed to limited access to treatment of cardiac disease in rural Eastern Cape compared to urban settings in Johannesburg, South Africa.

#### Mode of delivery and indications for caesarean section

Similar to the study done at a tertiary hospital in Johannesburg, South Africa, Balieva et al.^1^ reported a caesarean section rate of 66.7%, and the rate in the current study was quite high at 72.3%. This is so despite various guidelines recommending that women with cardiac disease deliver through spontaneous vaginal delivery.^32^ However, in both studies, the most common indications for caesarean births were not cardiac but obstetric causes.

The caesarean section rate in our study contrasts markedly with that reported in the NORMANDY study in Canada, which reported a rate of 37%, and another study conducted by Sliwa et al.^28^ in Cape Town, South Africa, which found a rate of 30%. The difference may be accounted for by the higher resource setting, better optimisation of cardiac disease before conception in early pregnancy and lower prevalence of RHD and PPCM in Canada and at Groote Schur Hospital in Cape Town, South Africa. In addition, the observation of greater obstetric indications for caesarean section than maternal cardiac complications may be explained by the effects of maternal cardiac dysfunction, which predisposes to ominous changes in placenta perfusion and function, nutrient and oxygen transfer to the foetus, resulting in abnormalities in foetal growth, hypoxia and subsequently promoting premature delivery.^33^

#### Adverse maternal outcomes

Almost 70% of the participants in the current study experienced complications, and over half of the women admitted into ICU had PPCM. The most frequently occurring complications were pulmonary oedema and severe pulmonary hypertension. The extremely high proportion of women who experienced complications was due to the delay in diagnosis. Cardiac disease in pregnancy is a leading cause of maternal mortality as it accounts for approximately 10%–16% of global maternal demise.^12^ In the current study, the case-fatality was 4.9%. However, in a study done in South Africa, the maternal case-fatality rate was reported as 3%.^8^ Our results contrast sharply with those from the study in Western Kenya that reported a maternal mortality rate of 9.3%^6^ and a much lower rate of 1.4% reported from the study done at an urban tertiary hospital in Johannesburg, South Africa.^1^

The maternal case-fatality rate of 4.9% observed in our study is clinically important and likely reflects several health system challenges characteristic of rural Eastern Cape. Nearly two-thirds of women were diagnosed only during the index pregnancy, while almost 40% received their diagnosis only after delivery, indicating substantial delays in recognition of cardiac disease. Furthermore, approximately one-quarter of participants were unbooked for antenatal care, and those who booked did so relatively late, limiting opportunities for early risk stratification and specialist intervention. Most patients were referred from district hospitals where access to echocardiography, specialist obstetric physicians, cardiologist and cardio-obstetric multidisciplinary teams is limited. Consequently, many women reached the tertiary centre only after developing advanced cardiac decompensation, as reflected by the high proportion presenting in NYHA functional class III or IV.

Geographic factors may have further contributed to adverse outcomes. Nelson Mandela Academic Hospital serves a large predominantly rural referral area across O.R Tambo, Alfred Nzo and neighbouring districts, where inter-facility referral delays may postpone access to definitive specialist care. Although ICU services were available at our institution, only 12.1% of women required ICU admission, suggesting that patients generally reached tertiary care after significant disease progression rather than benefiting from early referral and optimisation. The predominance of pulmonary oedema and pulmonary hypertension among women who died further supports the impact of delayed diagnosis and referral. Strengthening early cardiovascular screening during antenatal care, improving referral pathways and expanding multidisciplinary cardio-obstetric services at regional hospitals may therefore reduce preventable maternal deaths in this setting.

Surprisingly, the study done in an urban hospital in Cape Town found a comparable mortality rate to the current study of 5.9% despite different cardiac disease profiles in the study settings.^28^ These observations could be partially explained by the improved access to ICU and critical care services in South Africa compared to Western Kenya and by other factors such as differences in the prevalence of more severe disease. It is noteworthy that 13/22 (59.1%) of the participants who required ICU due to severe disease were discharged in a stable state.

### Perinatal outcomes

#### Assessment of neonatal presentation at birth

Most of the neonates had a 5-min Apgar score of ≥ 7, normal birthweight and were stable at birth, thus consequentially discharged. Our results are comparable to those from Western Kenya, where most of the neonates had either a 1-min, 5-min or 10-min Apgar score of ≥ 7 (90.3%); normal birthweight (69.6%); and were discharged after birth (76.2%).^6^ Similar results were reported from the tertiary hospital in Johannesburg: a 5-min and 10-min Apgar score of ≥ 7 (91.9% and 96.8%, respectively) and a normal mean birthweight of 2705 g ± 687 g^1^; as well as from the tertiary hospital in Cape Town, South Africa: Apgar score at 5-min was largely ≥ 7 (87.5%).^28^

These seemingly favourable neonatal outcomes in our study and also reported in other studies may be attributed to a high proportion of women with cardiac disease who, when diagnosed and initiated on appropriate multidisciplinary specialist care at tertiary hospitals, are able to carry their pregnancies beyond 34 weeks of gestation. According to various authors, careful management of women with cardiac disease enables them to tolerate pregnancy successfully.^34,35^ In addition, neonatal ICU services at tertiary institutions further contribute to improved foetal outcomes of neonates born prematurely. In the current study, all the neonates admitted to the neonatal ICU were discharged in a stable condition.

### Public health implications

From a health system perspective, these findings highlight the importance of strengthening integrated cardio-obstetric care in resource-limited settings. Earlier cardiovascular screening during antenatal care, improved referral pathways from district hospitals, expansion of echocardiography services, improved access to secondary rheumatic fever prophylaxis and timely referral for valve intervention before pregnancy may reduce maternal morbidity and mortality. Establishing dedicated multidisciplinary cardio-obstetric clinics could facilitate coordinated management of women with known or suspected cardiac disease throughout pregnancy and the postpartum period.

### Limitations

The findings of this study should be interpreted in light of several limitations. The study included only echo-confirmed cardiac disease, which likely excludes mild disease, undiagnosed disease and patients dying before echocardiography; therefore, prevalence may be underestimated. Again, our study design may not have sufficiently enabled us to conduct comparative analyses on our data. Prospective studies with more comprehensive data collection, especially regarding clinical signs and symptoms on presentation, would be of value in contributing to the development of future guidelines. Cardiac disease in pregnancy is rare; therefore, a case-control study design may have been more appropriate than a retrospective cross-sectional study design.

The study was conducted at a single tertiary referral hospital, which may limit the generalisability of the findings to other regions of South Africa, particularly urban centres or low-level healthcare facilities with different patient populations and referral pathways.

Several potentially important variables were unavailable in the medical records. In particular, HIV status was not consistently documented and therefore could not be analysed. Given the high prevalence of HIV in South Africa and its reported association with myocardial dysfunction and PPCM, the inability to evaluate its contribution represents an important limitation. Similarly, socioeconomic variables such as educational attainment, employment status, household income and other indicators of social deprivation were unavailable, precluding assessment of their influence on disease presentation, healthcare access and maternal outcomes.

Finally, because cardiac disease in pregnancy is relatively uncommon, the number of maternal deaths was small, limiting statistical power for identifying independent predictors of mortality. Consequently, multivariable analyses for mortality were not feasible. Future multicentre prospective studies with large sample sizes and more comprehensive clinical and socioeconomic data collection are needed to better identify predictors of adverse maternal and perinatal outcomes and to inform targeted interventions.

### Recommendations

The study provides the following recommendations:

- Paediatric services should prioritise prevention and early treatment of rheumatic fever and screening and early management of RHD among affected children.
- Contraception pre-pregnancy counselling should be offered to all patients with cardiac disease, and pregnancy should only be considered when the maternal condition is optimal.
- Correct diagnosis as early as possible in pregnancy is absolutely vital to improve maternal and foetal outcomes.
- There is a need for establishment of joint obstetric-cardiology clinics at NMAH.

## Conclusion

The prevalence was constant with rates reported from similar LMIC settings. However, cardiac disease in pregnancy was associated with severe maternal morbidity and relatively high mortality. Rheumatic heart disease and PPCM were the common cardiac lesions and major contributors for maternal and perinatal morbidity and mortality. Early diagnosis and multidisciplinary management were associated with good maternal and perinatal outcomes. Of greatest concern was the late stage at which these women were diagnosed. As such, there needs to be directed efforts towards routine screening for cardiovascular disease in pregnant women at referring institutions to ensure timely diagnosis, management and surveillance to promote optimal maternal and perinatal outcomes.
